# Clinical trial protocol for continuous glucose monitoring in critical care at Hospital Clinic of Barcelona (CGM‐UCI23)

**DOI:** 10.1111/nicc.13198

**Published:** 2024-10-28

**Authors:** Marc Pañero‐Moreno, Eva Maria Guix‐Comellas, Alberto Villamor‐Ordozgoiti

**Affiliations:** ^1^ Critical Care Nurse Hospital Clinic of Barcelona Barcelona Catalonia Spain; ^2^ Barcelona Clinical Research Foundation ‐ August Pi i Sunyer Biomedical Research Institute (FRCB‐IDIBAPS) Barcelona Catalonia Spain; ^3^ PhD Programme in Medicine and Translational Research University of Barcelona Barcelona Catalonia Spain; ^4^ Department of Fundamental and Clinical Nursing University of Barcelona Barcelona Catalonia Spain; ^5^ General Nursing Supervisior Hospital Clinic of Barcelona Barcelona Catalonia Spain

**Keywords:** continuous glucose monitoring, critical care nursing, critical illness, hyperglycaemia, intensive care

## Abstract

**Background:**

Hyperglycaemia is common in intensive care units (ICUs), with a prevalence of up to 86.2%, increasing mortality. Technology has evolved towards continuous glucose monitoring (CGM), and its use in ICUs began especially during the coronavirus pandemic (COVID‐19). Various studies have evaluated the reliability of CGM, indicating that it is safe for use in critically ill patients.

**Aim:**

The aim of this study was to compare the use of CGM with point‐of‐care glucose (POC‐G) testing in ICU. Specific objectives include evaluating the glycaemic control, the frequency of POC‐G measurements, the incidence of hyperglycaemia, hypoglycaemia and morbidity and mortality at 90 days.

**Study Design:**

An experimental, controlled and randomized clinical trial with a single‐blind design will be conducted at Hospital Clinic of Barcelona (HCB). A sample size of 376 participants will be recruited and randomly assigned to two groups: an experimental group, where glycaemic management will be based on CGM; and a control group, where glucose will be managed through POC‐G testing, with a blinded CGM.

**Results:**

The primary variable considered will be time in range (TIR), with secondary outcomes including, time above range (TAR), time below range (TBR), number of POC‐G measurements, incidence of hyperglycaemia and hypoglycaemia, and mortality. Hypothesis testing will use the Kolmogorov–Smirnov test to assess data normality, with appropriate statistical tests applied, considering a *p*‐value <.05.

**Relevance to Clinical Practice:**

The results obtained will help us understand the impact of CGM on critically ill patients. CGM could potentially reduce the workload of nurses and improve the efficiency of decision‐making by the ICU team, enabling early identification and treatment of glucose complications, thereby enhancing safety. Patient safety, a reduction in patient fingerstick and a decreased care burden are the criteria that add value to this research.

**What is known about the topic**

- Hyperglycaemia is prevalent in intensive care units (ICUs), affecting up to 86.2% of patients and increasing mortality risk.
- Continuous glucose monitoring (CGM) technology has advanced, especially during the COVID‐19 pandemic, and has been evaluated for reliability in critically ill patients.
- Although CGM shows promise in improving glycaemic control and reducing invasive blood tests, existing studies are limited by small sample sizes and during the COVID‐19 pandemic.

**What this paper adds**

- Further evaluation of CGM's impact in critically ill ICU patients is necessary, requiring more clinical trials.
- This study will compare the effectiveness of CGM with point‐of‐care glucose testing in managing glucose levels in an ICU setting.
- It aims to assess whether CGM can better manage glycaemia, reduce hyperglycaemia and hypoglycaemia episodes, and improve outcomes such as morbidity and mortality within 90 days post‐ICU discharge.
- The research seeks to provide evidence supporting CGM as a standard ICU practice, potentially reducing health care provider workload and enhancing patient safety.

## 1 BACKGROUND

The care of critically ill individuals has undergone a substantial transformation; intensive care units (ICUs) have evolved into highly specialized facilities.^1^

Critically ill patients exhibit blood glucose levels >180 mg/dL (10 mmol/L)^2^ with a prevalence of 77.8%–86.2%,^3^, ^4^ all contributing to heightened morbidity, mortality and safety concerns.^5^, ^6^, ^7^

The gold standard for ICU care is capillary blood glucose measurement, but there are instances where arterial blood or central venous blood samples are employed. These diverse approaches fall under the category of point‐of‐care glucose (POC‐G). Critically ill patients require three to six POC‐G measurements per day. In cases of intravenous insulin infusion, the frequency of POC‐G measurements may increase.^2^

Glucose control technology has advanced to continuous glucose monitoring (CGM), offering frequent measurements and their trends. Although CGM holds theoretical promise in improving hypoglycaemia detection and management compared with traditional POC‐G, it is important to note that it has not received approval from the U.S. Food and Drug Administration for inpatient use. However, during the COVID‐19 pandemic, the first studies appeared indicating that CGM can significantly enhance glycaemic management.^2^ CGM systems offer the opportunity to remotely monitor patients, providing alerts and the chance to prevent hypoglycaemia.^8^, ^9^

During the COVID‐19 pandemic, Agarwal et al. emphasized the feasibility, acceptability and reliability of CGM as a complementary measurement to POC‐G testing. A notable decrease in the frequency of POC‐G measurements, up to 71%, was observed when CGM was employed.^10^, ^11^, ^12^, ^13^, ^14^, ^15^

For CGM to be commercialized for people with diabetes in outpatient settings, a mean absolute relative difference (MARD) of less than 10% is required.^16^ However, in the ICU, an MARD below 15% is considered accurate, although a consensus is needed. Additionally, the Clarke Error Grid requires 98% of values to fall within zones A and B.^17^

Analysed articles found that CGM is accurate in ICUs with an MARD of 9.3%–13.9%, even during extracorporeal membrane oxygenation.^12^, ^13^, ^14^, ^18^, ^19^, ^20^, ^21^ The Clarke Error Grid results showed that 98.0%–99.7% of CGM readings fell within safe zones.^13^, ^14^, ^19^

Effective glycaemic control is indicated by a time in range (TIR) above 50% and a time below range (TBR) of less than 1%.^22^ Some authors reported a TIR of 46.1%–72% with a TBR of 0.25%–0.6%, reflecting excellent control and minimal hypoglycaemia.^10^, ^12^, ^13^, ^15^, ^23^

Although the results are promising, larger randomized controlled trials are needed to confirm the observed benefits.

## 2 AIMS

We aimed to compare the use of CGM with POC‐G in the ICU. Specific objectives include glycaemic control, frequency of POC‐G measurements, incidence of hyperglycaemia, hypoglycaemia, morbidity and mortality at 90 days.

## 3 DESIGN AND METHODS

An experimental, prospective, controlled, randomized and single‐blind clinical trial will be conducted at HCB. Unblinding will be permitted in case of adverse effects. Recruitment will take place from June 2024 to June 2026. This protocol was developed in accordance with the Standard Protocol Items: Recommendations for Interventional Trials (SPIRIT)^24^ and the World Health Organization Trial Registration Data Set.^25^

### 3.1 Setting and sample

HCB has 62 ICU beds and 47 intermediate care beds, totalling 109, distributed across 6 specialized ICUs, with an annual population of 13 262. The sample size was calculated using the approximation of two independent proportions via the GRANMO Sample Size Calculator (https://apisal.es/GRANMO). Accepting an alpha risk (*α*) of .05 and a beta risk (*β*) of .2 in a two‐tailed test, a sample size of 376 individuals is deemed necessary. The expected proportion for the control group in terms of TIR is 35%, while for the experimental group, it is assumed to be 50%. An estimated loss of 10% has been considered. The ARCSINUS approximation was used.

#### 3.1.1 Participants

Recruitment will occur during the ICU stay. Potentially eligible patients will be identified by the nursing team. As appropriate, the researcher will provide written and verbal information about the study and invite the patient to ask questions. For patients unable to sign the informed consent, a family substitute will be approached.

#### 3.1.2 Inclusion and exclusion criteria

Eligibility criteria include patients aged 18 years or over, having a stay in ICU and/or intermediate care, and having blood glucose levels >180 mg/dL (10 mmol/L). Exclusion criteria are pregnancy, individuals with contraindications for the use of CGM (such as skin issues or allergies to adhesives) and end‐of‐life process.

#### 3.1.3 Randomization

Simple random probabilistic sampling will be done in a 1:1 ratio to the experimental (CGM) or control group (POC‐G), creating two parallel groups. To reduce bias and improve the enrolment, randomization will be performed at the bedside by the nurses responsible for the patients. In the ICUs of HCB, a random, opaque and sealed envelope will be selected, assigning the participant to the corresponding control or experimental group.

### 3.2 Nurse training

The ICU nurses will receive training on CGM, including how to insert and configure the sensor before starting the trial. The training and improvement of the adherence to the protocol will be provided by the principal investigator, an expert in Diabetes Education and has been trained by the manufacturer of the CGM Dexcom G7 (Dexcom, San Diego, CA).

### 3.3 Intervention

This study consists of a comparison of two randomized groups:

- Experimental group: Critically ill patients admitted to the ICU with hyperglycaemia using CGM, as the basis for glycaemia management decisions.
- Control group: Critically ill patients admitted to the ICU at TH with hyperglycaemia using POC‐G, as the basis for glycaemia management decisions.

Both groups will have a Dexcom G7 CGM sensor. Each sensor requires a 30‐min warm‐up period. The Dexcom G7 CGM averages glucose values every 5 min, obtaining a total of 288 glucose readings over 24 h, ranging from 40 to 400 mg/dL (2.2–22.2 mmol/L). The sensor will be placed on the upper arm or abdomen. If a computerized tomography (CT) scan is needed, the CGM can be used without issue. However, in the case of magnetic resonance imaging (MRI), the sensor must be removed. The duration of each sensor's use will be a maximum of 10 days, but will end upon the patient's discharge, the need for MRI or in the case of death.

#### 3.3.1 Blinding

The patients involved in the study will be blinded, meaning they will not know whether they are in the experimental group using real‐time CGM or in the control group. The blinding process will be straightforward: both groups will have the same receiver and sensor inserted. In the experimental group, nurses will be able to see glucose values displayed on the receiver, while in the control group, the receiver will not show any values, effectively functioning as a ‘placebo’ sensor. This set‐up ensures that the patients cannot distinguish between the groups. Only the nurses will be aware of the group assignments, while the integrity of patient blinding is maintained.

#### 3.3.2 Control group

The CGM in the control group will be blinded, connected to the Dexcom G7 receiver to save the data, which will be downloaded later to the Clarity platform (Dexcom) for retrospective analysis.

POC‐G will be performed according to the usual protocol of TH. In case of glycaemic decompensation, POC‐G will be performed every 6 h and/or as needed. For patients with continuous intravenous insulin infusion, POC‐G may be performed every 2 h, 1 h or even every 15 min, depending on the stability of glucose levels and the nurse's judgement.

Therefore, there will be no intervention that modifies the usual treatment of patients in the ICU. The glucometer used in the centre is CareSens® Dual (Pascual y Furio Diabetes, Paterna, Valencia).

#### 3.3.3 Experimental group

The Dexcom G7 will be connected to the Dexcom G7 receiver, serving as an external monitor for nurses to use the data in real time. The data will be downloaded to Clarity. The following alarms will be set:

- Glucose level at 220 mg/dL (12.2 mmol/L).
- Glucose level at 80 mg/dL (4.4 mmol/L).
- Trend arrows.
- Upcoming urgent hypoglycaemia: An alert that notifies whether glucose is predicted to be <55 mg/dL (<3 mmol/L) in 20 min.
- Glucose level at <55 mg/dL (<3 mmol/L).

Additionally, the clinical team can perform as many POC‐G tests as they deem necessary. If the CGM values do not match the POC‐G values, the CGM can be calibrated. It should be noted that the comparison of POC‐G and CGM cannot be considered if the POC‐G was obtained during a rapid glucose change. Calibration can only be done when the patient's glucose is stable, when there is no active insulin (more than 3 h have passed since the last administration) and when there are no trend arrows.

#### 3.3.4 Safety criterion in experimental group

This protocol defines hypoglycaemia as a glucose level <80 mg/dL (<4.4 mmol/L), while it is usually defined as <70 mg/dL (<3.9 mmol/L) in individuals with diabetes. To prevent hypoglycaemia, we have set the threshold at <80 mg/dL (<4.4 mmol/L). Hyperglycaemia is defined as any glucose level >180 mg/dL (>10 mmol/L). For our clinical trial, patient safety is the top priority, so we have established the following safety criteria:

- *Values outside the measurement range*: The measurement range of the CGM is 40–400 mg/dL (2.2–22.2 mmol/L). Glucose levels outside this range must be confirmed with a POC‐G value.
- *Episodes of hyperglycaemia between insulin doses*: If glucose levels exceed 300 mg/dL (16.6 mmol/L) between insulin doses, and it has been more than 3 h since the last insulin dose, the nurse may alert the medical team to determine whether to administer additional insulin or wait until the next scheduled dose.
- *Episodes of hypoglycaemia*: If a value <80 mg/dL (<4.4 mmol/L) is detected, confirmation will be sought through a POC‐G measurement, and the ICU's standard protocol will be followed. This involves administering 10–15 g of intravenous or oral glucose, according to the patient's needs. A new POC‐G measurement will be taken after 15 min. If the POC‐G values recover and the CGM values are accurate, CGM values can be used.
- *Intravenous insulin*: During the administration of intravenous insulin, POC‐G control will be performed when glycaemia is <180 mg/dL (10 mmol/L), with trend arrows (↓↓) to confirm the accuracy of CGM values as a safety criterion. In cases of glycaemic stability, CGM values can be used.

The ICU team's decisions will always be considered, and the decision‐making process of the team will be analysed. In case of adverse effects, we will inform the ethics committee.

### 3.4 Data collection tools and methods

With consent, data will be collected from health records during the ICU stay and until 90 days post‐discharge. The CGM data will be stored on the Clarity platform, and the results will be cross‐referenced with the clinical records in the SAP (System Analysis Program Development) (SAP SE, Walldorf, Germany) of the HCB. Patient anonymity will always be maintained. All data will be analysed according to the intention‐to‐treat principle. Promoting participant retention is not necessary, because their participation is only during hospital admission.

The data will be coded, with each patient being assigned a number from 001 to 376. Each number will be associated with the patient's medical record number, creating a separate database for the information to be analysed. Only the principal investigator, who is responsible, will have access to this information, maintaining the relationship between each code and the patient's medical record number.

The secure storage and transfer of these data will be done through OneDrive (Microsoft Corp., Redmond, WA), included in the software of the HCB. The data collected will be kept for at least 5 years after completion. Subsequently, personal information will only be retained by the hospital for health care purposes and by the principal investigator for other scientific research purposes. Clarity, OneDrive and SAP will be protected with password.

Given the known safety profile of the device used, the highly controlled environment of the ICU and the ability of the clinical team to respond immediately, it is concluded that a Data Monitoring Committee is not necessary for this trial.

### 3.5 Data analysis

All collected information will be represented in contingency tables and graphs for the epidemiological analysis of the participating population. These representations will also be used for comparisons between population groups to demonstrate significant differences between the two groups concerning the specific variables proposed. The final decision to terminate the trial rests with the principal investigator.

#### 3.5.1 CGM versus POC analysis

Variables to be considered include glucose measures such as mean glucose, various percentiles (25, 50, 75), interquartile range, glucose management indicator, coefficient of variation, mean amplitude of glycaemic excursions, mean glucose at different times of the day, mean of daily differences, CGM duration, TIR, time above range (TAR), TBR, number of POC‐G, maximum and minimum glucose levels, incidence of hyperglycaemia and hypoglycaemia, mortality, hospital readmissions and so forth.

For confounding outcomes, variables such as age, sex, height, weight, body mass index, diabetes history, glycosylated haemoglobin, pathological history, reason for admission, ventilatory support and its duration, needs for CT and MRI, duration of ICU and hospital stay, SOFA (Sequential Organ Failure Assessment) score, APACHE II (Acute Physiology and Chronic Health Evaluation II) score and need for hemodynamic support will be considered.

For this study, we define morbidity as the presence of illness or disease and its impact on a patient's health during the 90 days following ICU discharge. It includes various health conditions and complications that affect the quality of life or functional ability, which were not present before hospital admission. Additionally, mortality refers to death from any cause during hospitalization or within the 90 days following ICU discharge. If there are specific causes of mortality during the study, these will be analysed. Death may result from the underlying condition that led to ICU admission. Therefore, we aim to observe whether the use of real‐time CGM reduces morbidity and mortality in the experimental group.

#### 3.5.2 Statistical inference

To demonstrate significant differences between the two groups concerning the specific variables posed, a statistical analysis will be performed. First, the normality of the data will be checked using the Kolmogorov–Smirnov test. Based on the results, the most appropriate statistical tests will be used. For all tests, a *p*‐value <.05 (less than the significance criterion, *α* = .05) will be considered, and a confidence level of 95% will be established.

Data will be analysed using JAMOVI (version 2.5.6 or higher). Descriptive statistics will be presented as mean ± standard deviation for continuous variables. Continuous variables expected to follow a normal distribution will be compared between groups using Student's *t*‐test, and analysis of variance (ANOVA) will be used when analysing more than two groups or independent variables. For continuous variables that do not meet the normality assumption, the Mann–Whitney *U* test will be applied, as it is suitable for non‐parametric data.

Categorical and count variables will be analysed with Fisher's exact test for proportion comparisons. For variables with high counts, Poisson regression will be employed to provide a more nuanced analysis.

Morbidity and mortality will be evaluated using Kaplan–Meier survival analysis to estimate event probabilities over time, with differences between groups assessed through log‐rank tests to compare survival curves. Additionally, the analysis will address missing data using multiple imputation techniques to ensure the robustness and reliability of the results.

### 3.6 Ethical and research approvals

Ethics approval has been obtained from the Ethical Committee of HCB (Approval number: HCB/2023/0377) and the Bioethics Commission of the University of Barcelona (Institutional Review Board: IRB00003099/CER052429), covering all participating sites.

The devices and platforms used are validated with CE certification for use with people; however, they will be used in a different environment. Therefore, it will be reported to the Spanish Agency of Medicines and Medical Devices (AEMPS) via the NEOPS platform with No. 23‐0039. Informed consent will be obtained by the principal investigator, and the possibility of using the data for ancillary studies will be documented in the consent form.

## 4 DISCUSSION

The increasing focus on improving glycaemic management highlights CGM as a significant advancement in this domain. Preliminary data indicate that CGM can enhance glycaemic control, reduce the frequency of invasive blood tests and lower the incidence of glucose‐related complications, thereby improving patient outcomes. The strengths of this study include its prospective, multi‐site design, rigorous methodology and comprehensive data analysis using standardized, validated tools.

This research is expected to pave the way for the adoption of CGM as a standard practice in ICUs, improving the overall quality of care for critically ill patients by reducing the burden on health care providers and enhancing patient safety.

The implementation of CGM in ICU settings has shown substantial promise, particularly during the COVID‐19 pandemic. Agarwal et al. emphasized the feasibility and reliability of CGM as a complementary tool to POC‐G.^10^ Some studies reported significant reductions in invasive procedures like POC‐G.^11^, ^12^, ^13^

The findings of Lu et al. present compelling evidence on the utilization of CGM in critically ill patients. Their study revealed a significantly lower incidence of continuous renal replacement therapy in the CGM group (9.5% vs. 21.4%, *p* = .046).^23^ The experimental trial by Lu et al. serves as a good starting point for researching the use of CGM in the ICU.

Nevertheless, a limitation of the current evidence is that most studies were conducted during the COVID‐19 pandemic, presenting unique challenges and constraints. Future research should aim to validate these findings in non‐pandemic settings to explore the broader applicability and benefits of CGM.

Large‐scale randomized controlled trials are necessary to confirm its efficacy and establish comprehensive guidelines.

## 5 LIMITATIONS

The main limitation is the difficulty of obtaining informed consent before the insertion of the CGM because of the critical condition of the patient. To mitigate this limitation, informed consent can be requested from family members. Another potential limitation is the number of participants available or willing to participate. Therefore, motivating the participating nurses will be crucial to obtaining the maximum number of participants.

There is also a possibility of selection bias because of the random assignment of patients to groups, as there may be differences in patient characteristics that could affect the results. Additionally, external factors or uncontrolled variables may influence the study's results.

## 6 FUNDING

This project has been funded and co‐financed by the Official College of Nurses of Barcelona (COIB) as part of the Nurse Research Projects Grants (PR687/2024) and Catalan Society of Intensive and Critical Medicine (SOCMIC) as part of Best Research Projects Grants. It has received the Second Runner‐Up Award for Best Innovative Capsule barnaclinic+ from HCB. The project has also received a grant for the completion of PhD from the General Council of Official Nursing Colleges of Spain (CGE). The CGM sensors and receivers have been provided by the manufacturer Dexcom (HOS‐2023‐021), with a contract in place to ensure no financial exchange or conflicts of interest.

Additionally, Dexcom will not have any involvement in data handling, ensuring data integrity and objectivity.

## 7 DISSEMINATION POLICY

The main results of this protocol will be submitted to this journal and disseminated to the scientific community through conferences. Results will be shared regardless of whether they are favourable or unfavourable. The complete protocol and informed consent materials, as well as the coded dataset and statistical code, will be made publicly available and can be shared with the scientific community upon request to the principal investigator and with approval from the ethics committee.

## 8 IMPLICATIONS FOR PRACTICE

There is scientific evidence demonstrating the accuracy of CGM in critically ill patients compared with POC‐G, but no experimental evidence yet confirms improved glycaemic control. The results of this study will help understand CGM's impact on critically ill patients, potentially reducing nurses' workload and improving decision‐making efficiency. Enhanced patient safety, fewer punctures and a reduced caregiving burden are key benefits, providing a basis for future research on cost‐effective glycaemic management.

As indicated by advanced practice nurses in diabetes care, nurses are the primary reference in glycaemic control. Innovation in this field must be led by nurses. With their expertise and hands‐on involvement in patient care, nurses are best positioned to implement and utilize such innovations to improve clinical decision‐making. The integration of CGM in ICU protocols should be spearheaded by nurses and the multidisciplinary team.

### 8.1 Impacts

This protocol is expected to provide valuable insights. CGM could lead to more precise and timely glucose management, reducing hyperglycaemia and hypoglycaemia, thus enhancing patient safety. Additionally, the use of CGM may decrease the frequency of invasive blood tests and the workload for nursing staff. This study could pave the way for adopting CGM as a standard in ICUs, improving overall patient care and potentially lowering health care costs by minimizing glucose‐related complications.

## 9 CONCLUSIONS

The study aims to provide robust evidence on the efficacy and safety of CGM in critically ill patients compared with traditional POC‐G testing. The anticipated benefits include enhanced patient safety, reduced nursing workload and improved clinical decision‐making, thereby fostering a more efficient and effective care environment. The findings could serve as a foundation for future research and the implementation of CGM in ICUs, ultimately aiming to improve patient outcomes and quality of care.

## AUTHOR CONTRIBUTIONS

All authors contributed to the literature review, methodological design, manuscript preparation and editing. Data management is the responsibility of the principal investigator; a monitoring committee is not necessary.

## FUNDING INFORMATION

This project has been financed and co‐financed by the Official College of Nurses of Barcelona (COIB) as part of the Nurse Research Projects Grants (PR678/2024) and the Catalan Society of Intensive and Critical Care Medicine (SOCMIC) as part of Best Research Project Grants. The CGM sensors and receivers have been provided by the manufacturer Dexcom, with a contract in place to ensure that there is no financial exchange or conflict of interest (HOS‐2023‐021).

## ETHICS STATEMENT

Ethical approval has been obtained from the Ethics Committee of the Hospital Clínic de Barcelona (Approval number: HCB/2023/0377) and the Bioethics Committee of the University of Barcelona (Institutional Review Committee: IRB00003099/CER052429), covering all participating centres.

## PATIENT CONSENT STATEMENT

The principal investigator will obtain informed consent, and the possibility of using the data for ancillary studies will be documented in the consent form. Ethical approval has been obtained.

## Data Availability

The full protocol and informed consent materials, as well as the coded dataset and statistical code, will be made publicly available and may be shared with the scientific community at the request of the principal investigator and with committee approval.
